# RVDB-prot, a reference viral protein database and its HMM profiles

**DOI:** 10.12688/f1000research.18776.2

**Published:** 2020-09-07

**Authors:** Thomas Bigot, Sarah Temmam, Philippe Pérot, Marc Eloit

**Affiliations:** 1 Hub de Bioinformatique et Biostatistique – Département Biologie Computationnelle, Institut Pasteur, USR 3756 CNRS, Paris, France; 2 Pathogen Discovery Laboratory, Institut Pasteur, Paris, 75015, France; 3 École Nationale Vétérinaire d’Alfort, Maisons-Alfort, 94700, France

**Keywords:** virus, genomes, proteins, hmm, clusters, annotations, database

## Abstract

We present RVDB-prot, a database corresponding to the protein equivalent of the nucleic acid reference virus database RVDB. Protein databases can be helpful to perform more sensitive protein sequence comparisons. Similarly to its homologous public repository, RVDB-prot aims to provide reliable and accurately annotated unique entries, while including also an Hidden Markov Model (HMM) protein profiles database for distant protein searching.

## Introduction

Sequence assignation often uses similarity criteria to infer homology, and hence taxonomy and / or protein function. In order to search for this, similarity, reliable, accurate and comprehensive databases are required. When trying to characterize sequences present in a metagenomics sample, searching first for related sequences in a viral database can lead to identify rapidly a known virus (high identity between the query sequence and the one in the database), or identify potential new species (low identity with any known sequence). Such hits must be further characterized on more comprehensive databases to increase the robustness of taxonomic assignations.

In the specific field of viruses, several solutions are available but their ability to provide valid results is highly dependent on the goal of the study and on the available computer resources. Using a database with a high number of sequences, such as NCBI nr/nt may seem appropriate, but it implies an increased computation time and annotation quality is not always optimal. Similarly, UniProtKB ^1^ contains numerous viral sequences (4 497 049 in total, including 17 008 (0.38%) reviewed) that could, as for NCBI/nr, increase computation time when thousands of sequences have to be analyzed concomitantly, which is routinely practiced in metagenomics analyses. RefSeq, on the other hand, is generally better curated but contains only full-length genomes, which reduces the diversity of available sequences, and also rarely includes the latest discoveries. RefSeq contains 13 180 virus sequences. Other specialized databases provide only specific groups of taxa for specific purposes, for instance, virus families responsible for infectious diseases like HIV or influenza viruses.

Thus, the need for better, well-annotated and comprehensive public viral database that can be used for the identification of viruses by high-throughput sequencing led Goodacre *et al.* to propose their Reference Viral DataBase (RVDB) ^2^. This database consists of a collection of all currently known viral genomes and virus-related nucleic sequences retrieved from NCBI/nr or RefSeq and includes a specific, both manual and computational reviewing process, as well as four updates of the contents per year. The reviewing process eliminates a great quantity of unwanted non-viral sequences like: cloning vectors, endogenous sequences, sequences that were wrongly annotated as virus but were actually of cellular origin, etc. This high level of curation makes RVDB quite attractive for the virology research community and in fact, in June 2020, version 19.0 was released.

Since viral genomes mainly consist of coding sequences, the need for an equivalent reference database that provides the protein version of these sequences may prove quite advantageous.

Indeed, protein sequences are useful when searching for distant homologs: their substitution rates are much lower than nucleic sequences. Additionally, proteins can also be efficiently clustered according to their similarity, and the resulting clusters can then be used to build Hidden Markov Model (HMM) profiles in order to identify more evolutionary distant proteins. In fact, programs like HMMER ^3^ allow the building of HMM profiles from a multiple sequence alignment of proteins. This profile can then help recognizing proteins based on complex position-specific models of sequence conservation and evolution, and it does so in a more accurate way than if a classic sequence alignment is used.

Therefore, we propose a protein sequence version of RVDB whose update will be synchronized with the original nucleotide RVDB release. Here we describe the conversion from the nucleotide version of RVDB to the protein version RVDB-prot, as well as the clustering process leading to the HMM profiles.

## Methods

### Conversion from RVDB nucleic database to RVDB-prot

The current version of RVDB, v19.0 ^4^ consists of a collection of 3 084 319 nucleic sequences ^[Bibr ref-2]^. The accession numbers were extracted in order to gather the corresponding database entries in Genbank format. From these entries, the corresponding coding domain protein sequences, description, and protein accession numbers were automatically recognized and copied into the protein collection. The process relies on the amino-acid sequences and information provided initially in the nucleic entry annotations. The resulting protein file contains the nucleic sequence reference, for traceability purposes. The sequence names are formatted in the following way:

>acc|<p_bank>|<p_acc>|<n_bank>|<n_acc>|<descr[sp]>, where:

p_bank is the bank in which the protein can be found

p_acc is the accession number corresponding to the protein sequence

n_bank is the bank in which the original nucleotide sequence was found

n_acc is the original information found in the nucleic database

descr is the description of the protein sequence as found in the database entry

sp is the species name.

This process produces a 4 705 359 protein sequence file.

### Generation of HMM profiles

The HMM generation rationale was inspired from vFam (the database of HMM profiles built from all the viral proteins present in RefSeq, discontinued from 2014) ^5^, but was entirely re-coded as a Snakemake pipeline ^6^, using different tools for some key steps (clustering, alignment). The proteins sequences were clustered with a 100% identity criterion to remove duplicates using CD-Hit 4.7.0 ^7^. Then, the sequences were processed using Blast 2.2.26 ^8^ performing an all-against-all comparison. These comparisons allowed Silix 1.2.6 ^9^ (using default parameters) to define clusters of sequences according to their similarity. This step produced a text file in which each sequence was associated to one cluster. The information of each cluster (containing at least four sequences) was transformed into a fasta file containing all the sequences within the cluster. Then, sequences were aligned using Mafft 7.023 ^10^ in auto mode. The multiple sequence alignments were processed by HMMER 3.2.1 ^3^ (hmmbuild, using default parameters) in order to obtain the HMM profiles. The HMM profiles were finally grouped into a single file.

### Annotation of HMM profiles

**Figure 1.**
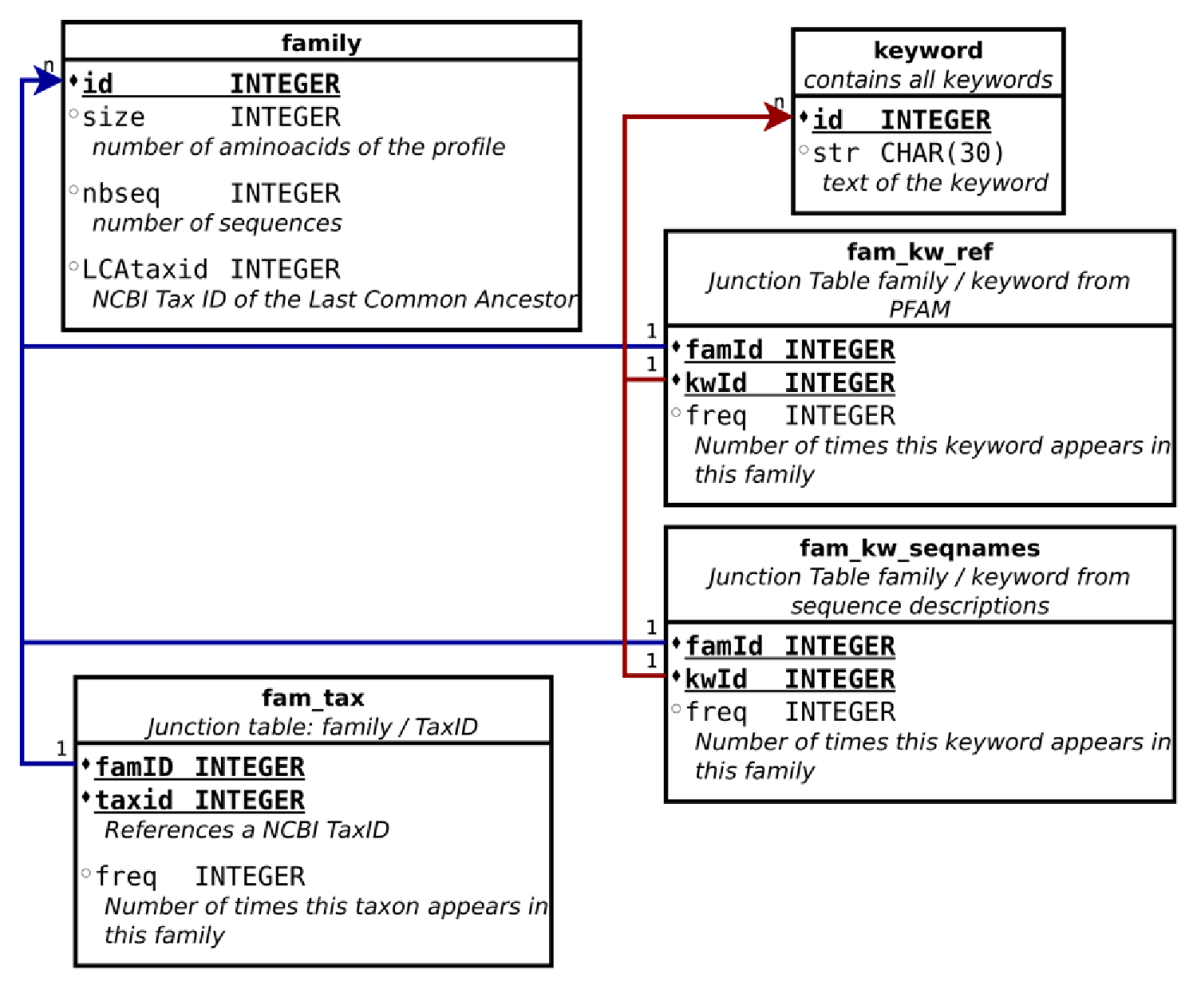
Schema of the annotation database.

A cluster is defined as a set of sequences, among which each sequence is characterized by its taxonomy ( *i.e.* a virus species) and eis associated with a description of its putative function, when it is known. In order to describe the different clusters, these information and other indicators (such as the cluster length and number of sequences) are combined into an annotation database, in SQLite format. The schema of this database is shown in Figure 1.

The first type of data associated to a cluster is a set of keywords describing the putative function of the proteins present in a given cluster. These keywords correspond to the union of all names of the significant sequences found in PFAM ^11^ (with --cut_ga parameter which tells HMMER to trust the cutoff defined by PFAM) using all the sequences of the cluster as queries, weighted according to their frequencies, and excluding trivial words. We also produce a complementary word frequency count using sequence descriptions. These keywords are stored separately from the PFAM ones. Despite the fact that sequence descriptions can be vague or inaccurate, they are a good fallback in case the cluster had no match with any PFAM one.

Here is an example of different keywords, using annotations from the cluster number 1, containing 18 sequences; the keywords and their frequencies from PFAM, are: RNA(58), viral(18), dependent(18), polymerase(18), helicase(17), cysteine(11), picorna(11); the first ten keywords from the sequence descriptions are: virus(17), protein(13), hypothetical(10), like(9), picorna(9), polyprotein(5), RNA(4), Wenzhou(4), Beihai(4), non-structural(2). Altogether, these keywords allow to describe a cluster composed of RNA-dependent RNA polymerase of picorna-like viruses. The complementarity of these two annotations is well illustrated here since the simple list of keywords would not have allowed to identify the function of this cluster (here the viral polymerase) without PFAM.

In addition to the protein description, the database stores the virus taxonomy associated to all the taxa, referring to tNCBI TaxIDs. For each cluster, the taxonomic information is summarized by a Last Common Ancestor (LCA) that corresponds to the taxon in the tree of life to which all the sequence taxa belong; this LCA can be close to the root of the tree (Viruses), but is usually specific to a family.

Finally, the database also provides the length (number of amino acids of the multiple sequences alignment) and the number of sequences in each cluster.

This database is available in SQLite format, and to provide more direct access, flat text files are proposed. A text file for each cluster, identified with its cluster number, contains all the information related to it.

## Software availability

The different steps explained above are performed using a Snakemake pipeline ^6^, available at Institut Pasteur’s Gitlab.

- Pipeline available from https://gitlab.pasteur.fr/tbigot/rvdb-prot/.
- Archived source code at time of publication: https://doi.org/10.5281/zenodo.4001989 ^12^
- Licence: GNU GPL v3.0

Several tools are needed to run the pipeline, including: Python, Mafft, Golden, HMMER, Snakemake, Silix, Blast+. The versions of these tools compatible with the pipeline are listed in the README file.

## Data availability

### Underlying data

Database files are available at https://rvdb-prot.pasteur.fr/. Release 19.0 described in this manuscript is also available from Zenodo.

Zenodo: U-RVDBv19.0 https://doi.org/10.5281/zenodo.4002051 ^4^.

This project contains the following underlying data:

- U-RVDBv19.0-prot.fasta (fasta file containing protein features of the original database: -prot.fasta)
- U-RVDBv19.0-prot.fasta-prot.hmm (the HMM profiles, generated with and for hmmer 3.2.1 (from 2019, 3.1b2 before))
- U-RVDBv19.0-prot.fasta-prot-hmm.sqlite (SQLite db containing annotations (please find a documentation below))
- U-RVDBv19.0-prot.fasta-annot.txt (a directory of annotations with plain text files (one per protein family))

Data are available under the terms of the Creative Commons Attribution 4.0 International license (CC-BY 4.0).

Table 1 shows some summary metrics for the entries of this release and the different resources.

**Table 1.** Metrics for release 19.0.

Nucleic sequences	RVDB	3 084 319
Proteins	RVDB-prot	4 705 359
Unique proteins	RVDB-prot	674 970
Clusters	RVDB-prot HMM	13 201

Updates are manually curated each time a new release of the main database (nucleic RVDB) is announced, i.e., four times a year. The following older versions are also available online: 18.0 (2020–03), 17.0 (2019–11), 16.0 (2019–06), 15.1 (2019–02), 14.0 (2018–09), 13.0 (2018–06), 12.2(2018–03), 11.5 (2017-–0), 10.2 (2017–04).

**Usage** HMMER can be used to search for all profiles in a fasta sequence file (sequences.fasta): hmmsearch U-RVDBv15.1-prot.fasta-prot.hmm sequences.fasta > result.out. Additional options are available in HMMER User’s Guide.
