# Supplementary material for: RVDB-prot, a reference viral protein database and its HMM profiles
Source: F1000Res. 2020 Sep 7;8:530. Originally published 2019 Apr 23. [Version 2] doi: 10.12688/f1000research.18776.2 (PMC7492780; doi:10.12688/f1000research.18776.2)
Supplement: Peer review history

# Peer review: Reviewer response for version 2

**DOI:** 10.5256/f1000research.29158.r70931

**Published:** 2020-09-15

**Authors:** Guy Perriere

**Affiliations:** 1 Laboratoire de Biométrie et Biologie Evolutive, CNRS, UMR5558, Universite Claude Bernard - Lyon 1, Villeurbanne, 69622, France

I have no other comments to add as the authors adressed all the concerns I raised in my previous review.

Are sufficient details of methods and materials provided to allow replication by others?

Partly

Is the rationale for creating the dataset(s) clearly described?

Yes

Are the datasets clearly presented in a useable and accessible format?

Yes

Are the protocols appropriate and is the work technically sound?

Yes

Reviewer Expertise:

Phylogeny, molecular evolution, comparative genomics, sequence databases

I confirm that I have read this submission and believe that I have an appropriate level of expertise to confirm that it is of an acceptable scientific standard.

# Peer review: Reviewer response for version 2

**DOI:** 10.5256/f1000research.29158.r70930

**Published:** 2020-09-07

**Authors:** Philippe le Mercier

**Affiliations:** 1 Swiss-Prot Group, CMU, Swiss Institute of Bioinformatics, Geneva, Switzerland

No further comments.

Are sufficient details of methods and materials provided to allow replication by others?

Partly

Is the rationale for creating the dataset(s) clearly described?

Yes

Are the datasets clearly presented in a useable and accessible format?

Yes

Are the protocols appropriate and is the work technically sound?

Yes

Reviewer Expertise:

Virus proteomics

I confirm that I have read this submission and believe that I have an appropriate level of expertise to confirm that it is of an acceptable scientific standard.

# Peer review: Reviewer response for version 1

**DOI:** 10.5256/f1000research.20570.r47713

**Published:** 2019-05-20

**Authors:** Guy Perriere

**Affiliations:** 1 Laboratoire de Biométrie et Biologie Evolutive, CNRS, UMR5558, Universite Claude Bernard - Lyon 1, Villeurbanne, 69622, France

This is an interesting resource that can be of use for people dealing with comparative genomics in viruses. There are some points that need to be clarified before this paper can be indexed though.

1. In order to ease reproducibility, the parameters used for the different programs (e.g. HMMER, SiLiX) of the pipeline should be provided.
2. Why is it required to locally translate the Coding DNA Sequences (CDS) from the original RVDB nucleotide database instead of downloading them from the resource?
3. I have a question for the taxonomic assignation to the Last Common Ancestor (LCA) when building the clusters. How are handled the possible contradictions within a cluster? More exactly, what is done exactly if sequences that belong to distantly related taxa are clustered together? If a strict LCA rule is applied, then it would be possible to have a really inprecise assignation (something like "virus" and that's it). 

Are sufficient details of methods and materials provided to allow replication by others?

Partly

Is the rationale for creating the dataset(s) clearly described?

Yes

Are the datasets clearly presented in a useable and accessible format?

Yes

Are the protocols appropriate and is the work technically sound?

Yes

Reviewer Expertise:

Phylogeny, molecular evolution, comparative genomics, sequence databases

I confirm that I have read this submission and believe that I have an appropriate level of expertise to confirm that it is of an acceptable scientific standard, however I have significant reservations, as outlined above.

# Response

**Published:** 2020-08-26

**Authors:** Thomas Bigot

**Affiliations:** Institut Pasteur, Paris, France

We would like to thank the Reviewer. Please find below our line-by-line responses.

**In order to ease reproducibility, the parameters used for the different programs (e.g. HMMER, SiLiX) of the pipeline should be provided.**

 Done. Parameters are now specified in the new version of the manuscript. Actually, for both of these programs, we use default parameters.

**Why is it required to locally translate the Coding DNA Sequences (CDS) from the original RVDB nucleotide database instead of downloading them from the resource?**

 We have clarified this point in the new version: actually, we use translations provided in the entry of each nucleic sequence. They are provided in the raw data of the original database (Genbank, RefSeq) along with the accession number. What we do amounts to retrieve all protein sequences

 corresponding to a nucleic sequence from protein database with accession numbers, but doing it directly from the nucleic database is faster.

**I have a question for the taxonomic assignation to the Last Common Ancestor (LCA) when building the clusters. How are handled the possible contradictions within a cluster? More exactly, what is done exactly if sequences that belong to distantly related taxa are clustered together? If a strict LCA rule is applied, then it would be possible to have a really inprecise assignation (something like "virus" and that's it).**

 Indeed, we use naïve LCA assignation, and it can lead to imprecise assignation (some clusters can be tagged as Viruses). As we do not have other information about the cluster we characterize, we chose not to avoid this possibility. We have added a precision about this case in the new version of the manuscript: “For each cluster, the taxonomic information is summarized by a Last Common Ancestor (LCA) that corresponds to the taxon in the tree of life to which all the sequence taxa belong; this LCA can be close to the root of the tree (Viruses), but is usually specific to a family.”

# Peer review: Reviewer response for version 1

**DOI:** 10.5256/f1000research.20570.r47715

**Published:** 2019-05-16

**Authors:** Philippe le Mercier

**Affiliations:** 1 Swiss-Prot Group, CMU, Swiss Institute of Bioinformatics, Geneva, Switzerland

In this article, the authors present a RVDB-prot, a reference viral protein database and its HMM profiles. The purpose of this approach is providing a complete reference database of viral proteins to identify new sequences. Their database is based on nucleotide Reference Viral Database (RVDB). The rationale of this work  is that protein sequences can be better than nucleotides for searching distant homologs.

 In brief, their approach was as follows: RVDB database was converted to proteins, thereby creating a new dataset of 3,899,699 proteins. The protein were clustered, and these clusters used to create HMM. Words frequently present  in sequence names of a cluster were used to annotate HMM profiles. The software, pipeline and final database are all available.

 The final data are of good quality, will hopefully  be maintained along with RVDB and offer a new approach for protein virus reference.

 While the article is well written and the method is well described, there are a number of issues that need to be addressed:

- The database is described as facilitating sequence assignation. This seems a bit vague, a sentence describing possible applications could help. 
-  The introduction may describe better the current state of research in the field. UniProtKB should be cited in “existing databases” for viral proteins, and authors may add citations of its use in virus detection. (ex: UniRef90 used with success to create synthetic human virome ^1^ (PMID: 26045439)). This would also highlight new potential applications for RVDB-prot.  
- UniProtKB provides data for 3,972,271 viral proteins, a bit more than RVDB-Prot (3,899,699). RVDB-prot data is based similarity gathering of sequences with viral RefSeq, which has the advantage to ignore any taxonomical issues. On the other hand, many RefSeq are provisional, and those are not free or errors. The authors may discuss the advantage of their method over existing protein dataset.
-  Similarly, UniRef90 contains 577,105 clusters of proteins, which could be compared to the 489,207 unique proteins of RVDB-prot. Further discussion may help understanding the advantages of these two datasets.  
- The paper could provide more details on parameters used for defining clusters with Silix.

 

 Minor remark:

 The naming system may be perfected. Although imaginative and automatic, it seems to be limited. For example cluster 77 in the -prot-hmm-txt.zip (v 15.1) contains 398 sequences, which are obviously rep proteins for ssDNA viruses circo, gemini and their satellites, but the names fished out by author’s method are not clear. This can be problematic for sequence assignation.

 Keywords for cluster 77:

 protein 329

 replication 296

 virus 183

 alphasatellite 154

 associated 138

 putative 120

 viral 103

 CRESS 78

 leaf 50

 curl 49

 initiator 49

 Rep 41

 yellow 39

 Circoviridae 39

 Actually, the name used to create RVDB-pro keywords is not clearly defined. The name of GenBank protein entry are not very consistent and this may explain these problems. Maybe using pfam or any other method of identification over the clusters may help naming them in a more consistent way.

Are sufficient details of methods and materials provided to allow replication by others?

Partly

Is the rationale for creating the dataset(s) clearly described?

Yes

Are the datasets clearly presented in a useable and accessible format?

Yes

Are the protocols appropriate and is the work technically sound?

Yes

Reviewer Expertise:

Virus proteomics

I confirm that I have read this submission and believe that I have an appropriate level of expertise to confirm that it is of an acceptable scientific standard, however I have significant reservations, as outlined above.

## References

[1] : Viral immunology. Comprehensive serological profiling of human populations using a synthetic human virome. *Science*.2015;348(6239) : 10.1126/science.aaa0698 aaa0698 10.1126/science.aaa0698 26045439PMC4844011

# Response

**Published:** 2020-08-26

**Authors:** Thomas Bigot

**Affiliations:** Institut Pasteur, Paris, France

We would like to thank the Reviewer. Please find below our line-by-line responses.

**The database is described as facilitating sequence assignation. This seems a bit vague, a sentence describing possible applications could help.**

 We have added the following sentence to exemplify possible applications: “When trying to characterize sequences present in a metagenomics sample, searching first for related sequences in a viral database can lead to identify rapidly a known virus (high identity between the query sequence and the one in the database), or identify potential new species (low identity with any known sequence). Such hits must be further characterized on more comprehensive databases to increase the robustness of taxonomic assignations.”

**The introduction may describe better the current state of research in the field. UniProtKB should be cited in “existing databases” for viral proteins, and authors may add citations of its use in virus detection. (ex: UniRef90 used with success to create synthetic human virome1 (PMID: 26045439)). This would also highlight new potential applications for RVDB-prot. UniProtKB provides data for 3,972,271 viral proteins, a bit more than RVDB-Prot (3,899,699). RVDB-prot data is based similarity gathering of sequences with viral RefSeq, which has the advantage to ignore any taxonomical issues. On the other hand, many RefSeq are provisional, and those are not free or errors. The authors may discuss the advantage of their method over existing protein dataset.**

 We have updated the introduction, introducing UniProtKB viral sequences: “UniProtKB11 contains numerous viral sequences (: 4 497 049 in total, including 17 008 (0.38%) reviewed ones) that could, as for NCBI/nr, increase computation time when thousands of sequences have to be analyzed concomitantly, which is routinely practiced in metagenomics analyses.” We have

 also updated the description of RefSeq (with updated contents) and better exemplified the benefit of RVDB over these two databases.

**Similarly, UniRef90 contains 577,105 clusters of proteins, which could be compared to the 489,207 unique proteins of RVDB-prot. Further discussion may help understanding the advantages of these two datasets.**

 We stressed on the first asset of RVDB: the curation that is done on the sequences is unique and allows to raise confidence in the fact that all the sequences of the database are real viral sequences.

**The paper could provide more details on parameters used for defining clusters with Silix.**

 Done. We used the default parameters of Silix.

**The naming system may be perfected. Although imaginative and automatic, it seems to be limited. For example cluster 77 in the -prot-hmm-txt.zip (v 15.1) contains 398 sequences, which are obviously rep proteins for ssDNA viruses circo, gemini and their satellites, but the names fished out by author’s method are not clear. This can be problematic for sequence assignation. Actually, the name used to create RVDB-pro keywords is not clearly defined. The name of GenBank protein entry are not very consistent and this may explain these problems. Maybe using pfam or any other method of identification over the clusters may help naming them in a more consistent way.**

 We are grateful for this remark which helped us make the naming system clear. Indeed, the pipeline does now query PFAM to annotate sequences. As explained in the new version of the manuscript, for each cluster, we query PFAM with every sequences of this cluster, using --cut_ga option of HMMER (this option makes HMMER trust PFAM GA bitscore cutoff defined for each cluster). We kept the original system (using sequences descriptions) despite the fact they are inaccurate, since sometimes, we do not find homologs clusters in PFAM.
